# Immunization dropout rate and data quality among children 12–23 months of age in Ghana

**DOI:** 10.1186/s13690-017-0186-8

**Published:** 2017-04-17

**Authors:** Benjamin Baguune, Joyce Aputere Ndago, Martin Nyaaba Adokiya

**Affiliations:** 1 Department of Community Health, School of Allied Health Sciences, University for Development Studies, Tamale, Ghana; 2 School of Hygiene, Environmental Health Programme, Ministry of Health, Box 88, Tamale, Ghana; 3 Department of Nursing, School of Allied Health Sciences, University for Development Studies, Tamale, Ghana

**Keywords:** Immunization, Dropout rate, Data quality, Techiman Municipality, Children, Ghana

## Abstract

**Background:**

Immunization against diseases is one of the most important public health interventions with cost effective means to preventing childhood morbidity, mortality and disability. However, a proportion of children particularly in Africa are not fully immunized with the recommended vaccines. Thus, many children are still susceptible to the Expanded Program on Immunization (EPI) targeted diseases. The objective of this study was to determine the immunization dropout rate and data quality among children aged 12–23 months in Techiman Municipality, Ghana.

**Methods:**

A cross-sectional cluster survey was conducted among 600 children. Data was collected using semi-structured questionnaire through face-to-face interviews. Before the main data collection, the tools were pre-tested in three different communities in the Municipality. The mothers/caregivers were interviewed, extracted information from the child immunization cards and observation employed to confirm the presence of Bacillus Calmette-Guerin (BCG) scar on each child. Routine immunization data was also extracted from immunization registers and annual reports in the Municipality.

**Results:**

**I**mmunization coverage for each of the fifteen vaccines doses is above 90.0% while full childhood immunized status is 89.5%. Immunization dropout rate was 5.6% (using BCG and Measles as proxy vaccines). This is lower than the 10.0% cutoff point by World Health Organization. However, routine administrative data was characterized by some discrepancies (e.g. > 100.0% immunization coverage for each of the vaccines) and high dropout rate (BCG - Measles = 31.5%). Binary regression was performed to determine predictors of dropout rate. The following were statistically significant: married (OR = 0.31; 95% = CI 0.15–0.62; and *p* = 0.001), Christianity (OR = 0.27; 95% CI = 0.13–0.91; and *p* < 0.001), female child (OR = 0.50; 95% CI = 0.26–0.91; and *p* = 0.024) and possession of immunization card (OR = 50.3; 95% CI = 14.40–175.92; and *p* < 0.001) were found to be associated with immunization dropout.

**Conclusion:**

Childhood full immunized status (89.5%) and immunization coverages (>90%) are high while dropout rate is lower than the recommended cutoff point by WHO. However, immunization data quality remains inadequate. Thus, health education and orientation of service providers is urgently needed. In addition, immunization registers and data quality are issues that require attention.

## Background

Immunization is the most effective means of combating communicable diseases [1]. It is proven as one of the most cost effective health interventions worldwide, through which a number of childhood diseases have been prevented or eradicated [2]. Immunization campaigns carried out from 1967 to 1977 by the World Health Organization (WHO) eradicated the natural occurrence of small pox. Currently, polio is eliminated from; America (in 1994), the Western Pacific (in 2000), and the European WHO regions (in 2002) [2]. Since the beginning of the Expanded Program on Immunization (EPI) in 1974, vaccines have significantly reduced vaccine preventable diseases (VPDs) and deaths worldwide. However, a proportion of children are not fully immunized with the recommended vaccines. Thus, many children are still susceptible to the EPI targeted diseases [3, 4]. In 2011, about 107 million infants (83%) worldwide received the third dose of Diphtheria-Pertussis-Tetanus (DPT) vaccine. Approximately, 22.4 million children failed (dropout) to receive the DPT3 dose leaving many children susceptible to VPDs and death [5]. In 2011, WHO estimated DPT3 coverage as 85% among children aged <12 months worldwide with a range of 71% in African region to 96% in western pacific [6]. Despite the global progress, many children particularly those in less developed countries like Ghana remain at risk of VPDs [4, 7].

In June 1978, Ghana launched the EPI with six antigens – Bacillus Calmette-Guerin (BCG), Measles, DPT and Oral Polio vaccine (OPV) for children under one year of age and tetanus toxoid (TT) vaccination for pregnant women. The launch was in response to the national health policy to reduce morbidity and mortality of VPDs which contributed significantly to both infant and child mortality in the country [8]. Fourteen years (1992) after the launch, the government added yellow fever vaccine to the national immunization program. In January 2002, Ghana in partnership with the Global Alliance for Vaccines and Immunization (GAVI) Initiative and supported by other health development partners added Hepatitis B and Haemophilus influenza type B vaccines. The two new vaccines are combined with DPT vaccine to form DPT-Hib-Hep (penta vaccine). Vaccines against Rotavirus Diarrhoea and Pneumonia diseases were also introduced in 2012. The EPI objective is to reduce the incidence of VPDs and poverty as well as the overall health system strengthening in the country. To achieve this objective, daily immunization services for children and pregnant women are provided through static and outreach sites [9].

Ghana Health Service (GHS) is committed to universal coverage of quality immunization services to all communities irrespective of geographical location and accessibility. According to the 2014 Ghana Demographic and Health Survey (GDHS), about 77% and 23% of children between the ages of 12 and 23 months were fully immunized and partially immunized (using immunization records and mothers’ recall) respectively. Moreover, infant and under five mortality rates were reported as 41 and 60 deaths per 1000 live births in 2014, 50 and 80 deaths per 1000 live births in 2008 and 64 and 111 deaths per 1000 live births in 203 respectively [10].

In Techiman, routine administrative immunization coverage ranges from 101.4% (Penta-3) to 167.6% (OPV-0) according to the Techiman Municipal Health Directorate (TMHD) report in 2015 [11]. While immunization dropout rate was 31.5% (using BCG and measles as proxy vaccines) [11]. In 2001, WHO and GHS indicated a dropout rate >10% in the EPI program requires an action [12]. Administrative coverage are inconsistent, abnormally high as well as high dropout rates [12]. Routine reports provide information on immunization coverage; however, these may be inaccurate and misleading. Therefore, coverage surveys can validate routine reports and provide additional information on immunization and identify strategies to improve immunization activities [12, 13]. Thus, the objective of this study was to determine the immunization dropout rate and data quality among children aged 12–23 months in Techiman Municipality, Ghana.

## Methods

### Study and setting

Techiman Municipal is situated in the central part of the Brong-Ahafo Region of Ghana. It shares boundaries with five districts, namely: Techiman North, Akumadan, Nkronza, Wenchi and Sunyani West Districts. The Municipality has a population of 166,497 projected from the 2010 population and housing census [11]. This population represents about 6.4% of the Regional total population. It has the highest population density of 256.5 people per square kilometer. Health services are provided through public and private health facilities. They include Health centres, Community-based Health Planning and Services Compounds (CHPS), Clinics and Maternity homes. As part of decentralization of the health system, the Municipality has been demarcated into seven sub-municipals to facilitate health services delivery.

### Study design

A cross-sectional cluster survey design was employed. All children between the ages of 12–23 months were eligible. Firstly, 30 clusters (communities) were selected in the Municipality. They were selected using probability proportional to their size (estimated population data). The community data was provided by the Municipal health directorate. The second stage involved the use of the EPI random walk method to select 20 children from each cluster. Two field enumerators were recruited and trained on the study protocol and semi-structured questionnaires. In addition, a pre-test was conducted after the training to determine the validity and reliability of the study tools. Actual data collection happened from 30^th^ January to 20^th^ February 2016.

### Sampling procedure

A '30 × 20' cluster sampling method was used for the study. The 30 clusters were selected using cluster identification form through the probability proportionate to size simple random method. Twenty (20) households from each of the 30 clusters were sampled. The starting point was selected as the first household for each cluster and then continued to the next nearest household until 20 eligible children were obtained. Door-to-door visits and face-to-face interviews were conducted with mothers/caregivers who had children 12–23 month as well as observation of the children for the presence of BCG scar.

### Sample size determination

The sample size was calculated using the formula N = [De × Z^2^ × p (1-p)]/d^2^ [14]. Where, N is the sample size, De (2) is the design effect, the ratio between the variance from the cluster design to the variance that would be obtained from a simple random sampling [14], Z (1.96) is the certainty wanted expressed in the percentage point of normal distribution corresponding to the 2-sided level of significant, P (77%) is the immunization coverage of Ghana [10] and d (5%) is the desired width of the confidence interval. Therefore; N = [2 × (1.96)^2^ × 0.77× 0.23]/ (0.05)^2^ = 545. A non response rate of 10% was added, giving a total sample of 600. Proceeding from house to house looking for the inclusion criteria of haven at least one child aged 12–23 months, 600 respondents (mothers and caregivers) were selected and interviewed.

### Data collection instrument and procedures

A modified WHO-EPI semi-structured questionnaire was used for the data collection. The questionnaire included items on socio-demographic characteristics and infant immunization information. After informed consent was received, the mothers/caregivers of selected children participated in a structured interview. Information on immunization coverage was obtained in two ways: immunization cards and mothers’/caregivers’ verbal reports. All mothers/caregivers were asked to show the interviewer the child health record card with immunization dates. If the card was available, the interviewer then extracted the dates of each immunization received. In cases where it indicated in the immunization card that the child did not receive all vaccines, the mother/caregiver was asked whether the child had received other vaccines that were not recorded on the card. If they answered yes, the information was recorded. If there was no card, or if the mother/caregiver was unable to show it to the interviewer, the child’s immunization information was based on their recall. Secondary data on routine immunization coverage was also extracted from registers and annual reports at the TMHD.

### Operational definitions

#### Fully immunized

Child received 1 dose of Bacillus Calmette-Guerin (BCG), 4 doses of Oral Polio Vaccine (OPV), 3 doses of Pentavalent, 3 doses of Pneumococcal Vaccine (PCV), 2 doses of Rotarix (Rota) and 1 dose of Measles and 1 dose of Yellow fever vaccines is said to be fully immunized. That is, a total of seven (7) vaccines and fifteen (15) doses.

#### Partially immunized

Child missed some of the prescribed vaccines doses considered to protect against vaccine preventable diseases.

#### Not immunized

Child received none of the prescribed vaccines doses considered to protect against vaccine preventable diseases.

#### Dropout rate

Percentage difference in coverage between two different doses in sequence.

### Data processing and analysis

At the end of the interviews, questionnaires were checked for completeness and internal consistency. Data was entered, cleaned and analyzed using Statistical Package for Social Sciences (SPSS) version 17.0. Descriptive statistics such as frequencies and percentages were produced and presented in tabular form. In addition binary regressions was also performed between dropout rate and socio-demographic characteristics of the respondent. Moreover, dropout rates between two vaccines doses in sequence were computed using the formula: Dropout rate = [(coverage of initial vaccine dose – coverage of ending vaccine dose) ÷ (coverage of initial vaccine dose) × 100], e.g. (BCG-Measles)/ (BCG)*100.

### Ethics approval and informed consent

An introductory letter and approval was received to conduct the study from the School of Allied Health Sciences, University for Development Studies, Tamale, Ghana. In addition, permission letter was obtained upon a written request and explanation of the study protocol, methods and questionnaire from the Techiman Municipal Health Directorate. At the individual level, the protocol, methods and approach was explained in English or Twi (main local language) and a written consent was obtained from each respondents of 18 years of age and above before the interview was conducted. Among the few teenagers, consent was obtained through their husbands (those married) or parents (those unmarried). Respondents were informed that participating was voluntary and it was their right to stop at any time. They were also informed of data confidentiality by not using any personal identifiers.

## Results

In total, 600 children aged 12–23 months and their mother/caregivers were recruited for the study. Nearly, one-fifth (18.5%) of the respondents had no formal education while 7.5% had formal education up to tertiary level. About three-quarters (67.5%) of the mothers were Christians and Muslims were 25.5%. More than half (52.0%) of the respondents were married. Overall, 9.5% were <19 years and only 5.5% were ≥ 50 years of age. Less than half (43.0%) of the respondents were traders and 10.5% reported as salary workers (Table 1).

**Table 1:** Socio-demographic characteristics of mothers/caregivers, in Techiman Municipality, Ghana, 2016

Variable		Number	Percentage (%)
Education	No formal education	111	18.5
	Primary school	126	21.0
	JSS/Middle school	252	42.0
	SHS	66	11.0
	Tertiary	45	7.5
Age	<19 years	57	9.5
	20–29 years	225	37.5
	30–39 years	219	36.5
	40–49 years	66	11.0
	≥50	33	5.5
Marital status	Never married	144	24.0
	Married	312	52.0
	Divorce	27	4.5
	Separated	114	19.0
	Widowed	3	0.5
Ethnicity	Akan	282	47.0
	Dagaati	102	17.0
	Frafra	57	9.5
	Kusaasi	66	11.0
	Ewe	39	6.5
	Dagomba	39	6.5
	Others^a^	15	2.5
Religion	Islam	153	25.5
	Christianity	405	67.5
	Traditionalist	6	1.0
	No Religion	36	6.0
Occupation	Salary worker	63	10.5
	Trader	258	43.0
	Farmer	132	22.0
	Artisan	99	16.5
	Housewife	48	8.0
Sex of child	Male	303	50.5
	Female	297	49.5
Card possession	Yes	582	97.0
	No	18	3.0
Relationship with child	Mother	540	90.0
	Caregiver	60	10.0

*Others* ^a^ *(Gurisi - 4, Krobo – 3, Wangara – 3, Sisaala – 3, Gruma – 1 and Fulani – 1)*

### Immunization status among children

Nearly, nine out of ten children (89.5%) were fully immunized at one year of age and above while 72.5% were fully immunized before one year of age, 9.5% were partially immunized and the remaining 1.0% received no vaccine (Table 2).

**Table 2:** Immunization status among children age 12–23 months in the Techiman Municipality, Ghana, 2016

Variable	Number	Percent (%)
Fully immunized by time of survey (one year and above)	537	89.5
Fully immunized before one year	435	72.5
Partially immunized	57	9.5
Not immunized	6	1.0
Total	600	100.0

### Comparison of survey and routine immunization dropout rates

The results show the proportion of children who received initial vaccine doses and dropped out before completing the schedule (15 doses) in the study. Dropout rate was determined for the entire EPI program using BCG as the entry vaccine and Measles as the exit vaccine (BCG coverage minus Measles coverage/BCG coverage*100). Immunization dropout rates for the multi-dose vaccines (OPV, Penta, PCV and Rotarix) were also calculated and compared with the routine immunization dropout rates in 2015. The routine immunization dropout rate (31.5%) are high in 2015 compared to 5.6% dropout rate in the present study. For the multi-dose vaccines, Rotarix had no dropout rate in this study versus 6.8% based on routine immunization reports in 2015. Penta dropout rate (5.1%) was the highest in the survey. While OPV dropout rate (39.2%) was the highest in the routine immunization report (Table 3).

**Table 3:** Comparison of routine immunization and survey dropout rate, in Techiman Municipal, Ghana, 2016

Vaccines in series	Routine dropout rate, 2015		Survey dropout rate, 2016		Absolute difference in dropout rate (%)
	Number	Percent (%)	Number	Percent (%)	
BCG – Measles	3358	31.5	33	5.6	25.9
OPV-0 – OPV-3	4361	39.2	18	3.1	36.1
Penta-1 – Penta-3	501	6.9	30	5.1	1.8
PCV-1 – PCV-3	1023	13.0	29	4.9	8.1
Rotarix-1 – Rotarix-2	544	6.8	0	0.0%	6.8

*BCG* Bacillus Calmette-Guerin

*OPV* Oral Polio Vaccine

*Penta* Pentavalent vaccine (DPT-Hib-Heb)

*PCV* Pneumococcal Vaccine

### Comparison of survey and routine immunization coverage

Immunization coverage from routine administrative data were higher compared to the survey findings for all the 15 vaccines doses. Coverage for the routine immunization ranges 101.4% (Penta-3) to 167.6% (OPV-0) and coverages among the survey results ranges from 92.0% (Measles and Yellow fever) to 99% (OPV-1, OPV-2, Penta-1, Penta-2, Rotarix-1 and Rotarix-2). Coverage of all the first doses of multi-dose vaccines are higher than the last in series, except Rotarix in the study (Table 4).

**Table 4:** Comparison of routine administrative immunization and survey coverages in Techiman Municipal, Ghana, 2016

Vaccine	Routine coverage, 2015		Coverage survey, 2016		Absolute % difference between the same vaccine
	Number of respondents = 6630		Number of respondents = 600		
	Number	Percent (%)	Number	Percent (%)	
BCG	10677	161.0	585	97.5	63.5
OPV-0	11114	167.6	582	97.0	70.6
OPV-1	7991	120.5	594	99.0	21.5
OPV-2	7347	110.8	594	99.0	11.8
OPV-3	6753	101.8	564	94.0	7.8
Penta-1	7250	109.4	594	99.0	10.4
Penta-2	6749	101.8	594	99.0	2.8
Penta-3	6723	101.4	564	94.0	7.4
PCV-1	7874	118.7	591	98.5	20.2
PCV-2	7296	110.1	591	98.5	11.6
PCV-3	6851	103.3	562	93.7	9.6
Rotarix-1	7990	120.5	594	99.0	21.5
Rotarix-2	7446	112.3	594	99.0	13.3
Measles	7319	110.4	552	92.0	18.4
YF	7619	114.9	552	92.0	22.9

*Nr* Routine immunization target population for 2015

*Ns* Survey sample size

*BCG* Bacillus Calmette-Guerin

*OPV* Oral Polio Vaccine

*Penta* Pentavalent vaccine (DPT-Hib-Heb)

*PCV* Pneumococcal Vaccine

*YF* Yellow Fever Vaccine

### BCG immunization and formation of BCG scar

The results on BCG immunization status and formation of scar are presented in Table 5. Majority (92.5%) the children who were immunized also had visible BCG scar (immunization status based on card - 95.3% versus mothers’ recall - 80.0%). The remaining 7.5% had no visible BCG scar (immunization status based on card – 4.7% versus mothers’ recall – 20.0%). All defaulted children did not have visible BCG scar (Table 5).

**Table 5:** BCG immunization status and BCG scar among children age 12–23 months in the Techiman Municipality, Ghana, 2016

		BCG SCAR		Total
		Yes	No	
BCG COVERAGE	Given (Card only)	543 (95.3%)	27 (4.7%)	570 (100.0%)
	Given (History)	12 (80.0%)	3 (20.0%)	15 (100.0%)
	Not given	0 (0.0%)	15 (100.0%)	15 (100.0%)
Total		555 (92.5%)	45 (7.5%)	600 (100.0%)

*BCG - BCG* Bacillus Calmette-Guerin

### Factors predicting immunization dropout

Table 6 show predictors of immunization dropout in the study area. In the binary multivariate analysis, factors found to be statistically significant with dropout were marital status, religion, sex of child and possession of immunization card. Children from married mothers (OR = 0.31, 95% CI = 0.15–0.62; and *p* = 0.001) are less likely to drop from the immunization schedule than children from unmarried. Also, children with Christian mothers (OR = 0.27; 95% CI = 0.13–0.91; and *p* < 0.001) are less likely to dropout immunization schedule compared to non-Christian. Male children are more likely to drop out compared to females (OR = 0.05; 95% CI = 0.26–0.91; and *p* = 0.024). Children without immunization cards (OR = 50.3;95% CI = 14.40–175.92; and *p* < 0.001) are more likely to drop out compared to those who possessed it.

**Table 6:** Binary regression of dropout rate and socio-demographic characteristics of respondents in Techiman Municipality, Ghana, 2016

Variables	OR (Univariate) (95% CI)	*P*-value	OR (Multivariate) (95% CI)	*P*-value
Age of respondent				
≤19 years	1.0		1.0	
>19 years	0.59 (0.27–1.27)	0.175	0.45 (0.18–1.14)	0.093
Relationship to child				
Mother	1.0		1.0	
Not mother	0.94 (0.39–2.29)	0.894	0.83 (0.32–2.17)	0.704
Parity				
1–2 children	1.0		1.0	
>2 children	1.31 (0.76–2.24)	0.319	1.38 (0.71–2.68)	0.342
Education status				
No formal education	1.0		1.0	
Formal education	0.70 (0.37–1.29)	0.253	0.97 (0.46–2.07)	0.946
Marital status				
Not married	1.0		1.0	
Married	0.66 (0.39–1.12)	0.127	0.31 (0.15–0.62)	**0.001**
Ethnicity				
Akan	1.0		1.0	
Not Akan	1.21 (0.71–2.04)	0.487	0.62 (0.31–1.27)	0.192
Religion				
Not Christian	1.0		1.0	
Christian	0.49 (0.29–0.83)	**0.008**	0.27 (0.13–0.91)	**<0.001**
Child sex				
Male	1.0		1.0	
Female	0.60 (0.35–1.02)	0.058	0.50 (0.26–0.91)	**0.024**
Immunization card possession				
Yes	1.0		1.0	
No	20.82 (7.50–57.82)	**<0.001**	50.3 (14.40–175.92)	**<0.001**

Bold data were statistically significant at 95% CI and *P* = 0.05

## Discussion

This study determined immunization dropout rate and data quality among children aged 12–23 months in Ghana. Overall, childhood full immunization status was 89.5% while coverage was 90.0% in the present study. This is about 12.5% higher than the national status (77.0%) [10]. Immunization coverage based on child immunization card and mothers’ recall for various vaccines were high - from 92.0% (Yellow fever and Measles) to 99.0% (OPV1, OPV2, Penta 1, Penta 2 and Rota). However, administrative data exceeded 100.0%. For example, 101.4% for Penta-3 and 167.6% for OPV-0 as reported in the 2015 [11]. These discrepancies and high coverages are unexplained except to speculate such as under qualified staff responsible for handling immunization data and inadequate facilitative supervision and monitoring. Another possible explanation is low utilization of data in planning and decision-making particularly at the sub-national levels. This may also be attributed to poor data management including inaccurate population denominator.

Even within the same routine administrative data, there are inconsistencies and problems of sequence. For instance, the findings show OPV-0 coverage (167.6%) to be even higher than BCG coverage (161.0%) from the routine administrative data. This is an unusual pattern and problem of immunization sequence. BCG vaccine is given up to one year of age while OPV-0 vaccine is given only up to two weeks of child’s life [9]. The expectation is for BCG coverage to be higher than OPV-O due to the longer duration and the opportunity to still take the vaccine up to one year compared to only two weeks period for OPV-0. Without any strong evidence for this discrepancy, we are attempted to attribute it to the observed immunization data management problems.

Though high immunization coverages are reported in the present study, there are still problems of dropouts in immunization services. In Ghana, dropout rate is determined using coverages of BCG as entry vaccine and Measles as exit vaccine. Dropout rates is a proxy measure of utilization of EPI services. In addition, it is an indicator of the health system capacity to deliver services that require multiple visits by clients and health service providers [15]. The dropout rate of 5.6% is lower than both the national rate (8.3%) and WHO recommended cutoff point (10.0%) [10, 12]. The present dropout rate in our study differs from previous studies conducted in Senegal (2005), Ghana (2015) and Nigeria (2005) where 30.9%, 31.5% and 65.5% were reported as dropout rates respectively [11, 16, 17]. This success of lower dropout rate is likely due to the continued decentralization efforts in the health system as well as additional establishment of Community-based Health Planning Services (CHPS). CHPS serves as an entry point which provides both static and outreach sites with overall improvement in access of immunization services and community involvement through health volunteers for defaulter tracing. One possible explanation for the high national level dropout rate may be underestimation of children qualified to receive immunization.

In the present study, predictors of dropout included marital status, religion, sex of child and possession of immunization card. Children from married families were less likely to drop out from immunization schedule compared to unmarried. Married families are more economically stable and most likely to discuss the health needs of their children including immunization. In addition, married women often receive addition support from their husbands and they feel confident to go out with their children for social and health programs. Unmarried mothers are unable to make healthy choices due to psychological trauma as well as hardships associated to unplanned single parenting.

Children from non-Christian families were more likely to drop out from immunization schedule compared to Christians. Religion remains a major factor in decision making including health seeking behavior. Sometimes, the actions and inactions of people from certain religions denomination are influenced by their perceptions rather than reality. This is a potential explanation for the findings of this study. Similar findings have been reported due to misconceptions by Muslim populations which negatively affected immunization up take in Northern Nigeria [18]. We did not verify the misconception among Muslim groups that, immunization is a deliberate strategy to reduce the Muslim population rather than prevention of diseases. The existence of such misconceptions has the potential to continue to affect immunization uptake.

This study found more males dropout from immunization. Though, similar relation has been reported in Ghana, more females dropout from immunization compared to their males [10]. Sex as a predictor of immunization dropout is reported in other studies [6, 19]. Kidane et al. (2008) [19] found that, in spite universal access efforts to immunization services, sex discrimination against female children continue to exist in rural areas of Bangladesh.

Possession of immunization card is a predictor of dropout. Children without cards have higher probability of dropout. This is similar to a study conducted by Russo et al. (2015) in the Dschang Region of Cameroon [20]. Irregular visits to health facilities by mothers can affect possession of immunization card. However, mothers who accidentally loss these cards may feel reluctant to visit health facilities for new immunization due to perceived negative attitudes of some health workers if they are unable to provide the card. This may lead to dropout. It could also mean that certain immunizations may have been given to those children but because there are no records, mothers could easily forget and recall that no immunizations were given.

These findings have policy implications for health system in the country. The discrepancies found in the routine administrative data may lead to low utilization of the information planning and decision-making. It may also lead to under budgeting and supply of vaccines which can have unbearable consequences during outbreaks or emergencies. However, this can be seen as an opportunity to address the existing gaps and improve the overall health system through immunization in the country.

### Limitation of the study

The study was limited due to recall bias. In addition, the comparison of 2015 routine administrative data versus 2016 survey data is inappropriate. However, the immunization card helped to valid the information. In addition, the administrative data gives an overall picture of the discrepancies, data quality and challenges within the health system which needs urgent attention.

## Conclusion

Childhood full immunized status (89.5%) and immunization coverages (>90%) are high while dropout rate is lower than the recommended cutoff point by WHO. However, immunization data quality remains inadequate. Thus, health education and orientation of service providers is urgently needed. In addition, immunization registers and data quality are issues that require attention.

## Abbreviations

BCG: Bacillus calmette-guerin
CHPS: Community-based health planning and services
EPI: Expanded program on immunization
GAVI: Global alliance for vaccines and immunization
GDHS: Ghana demographic and health survey
GHS: Ghana health service
JHS: Junior high school
OPV: Oral polio vaccine
PCV: Pneumococcal vaccine
SHS: Senior high school
TMHD: Techiman municipal health directorate
WHO: World Health Organization
